# Inhibiting DNA Polymerases as a Therapeutic Intervention against Cancer

**DOI:** 10.3389/fmolb.2017.00078

**Published:** 2017-11-21

**Authors:** Anthony J. Berdis

**Affiliations:** ^1^Department of Chemistry, Cleveland State University, Cleveland, OH, United States; ^2^Center for Gene Regulation in Health and Disease, Cleveland State University, Cleveland, OH, United States; ^3^Case Comprehensive Cancer Center, Cleveland, OH, United States

**Keywords:** DNA polymerases, chemotherapy, nucleoside analogs, DNA damaging agents, cancer

## Abstract

Inhibiting DNA synthesis is an important therapeutic strategy that is widely used to treat a number of hyperproliferative diseases including viral infections, autoimmune disorders, and cancer. This chapter describes two major categories of therapeutic agents used to inhibit DNA synthesis. The first category includes purine and pyrmidine nucleoside analogs that directly inhibit DNA polymerase activity. The second category includes DNA damaging agents including cisplatin and chlorambucil that modify the composition and structure of the nucleic acid substrate to indirectly inhibit DNA synthesis. Special emphasis is placed on describing the molecular mechanisms of these inhibitory effects against chromosomal and mitochondrial DNA polymerases. Discussions are also provided on the mechanisms associated with resistance to these therapeutic agents. A primary focus is toward understanding the roles of specialized DNA polymerases that by-pass DNA lesions produced by DNA damaging agents. Finally, a section is provided that describes emerging areas in developing new therapeutic strategies targeting specialized DNA polymerases.

## Biological roles of DNA polymerases

### DNA synthesis

**Figure 1:**
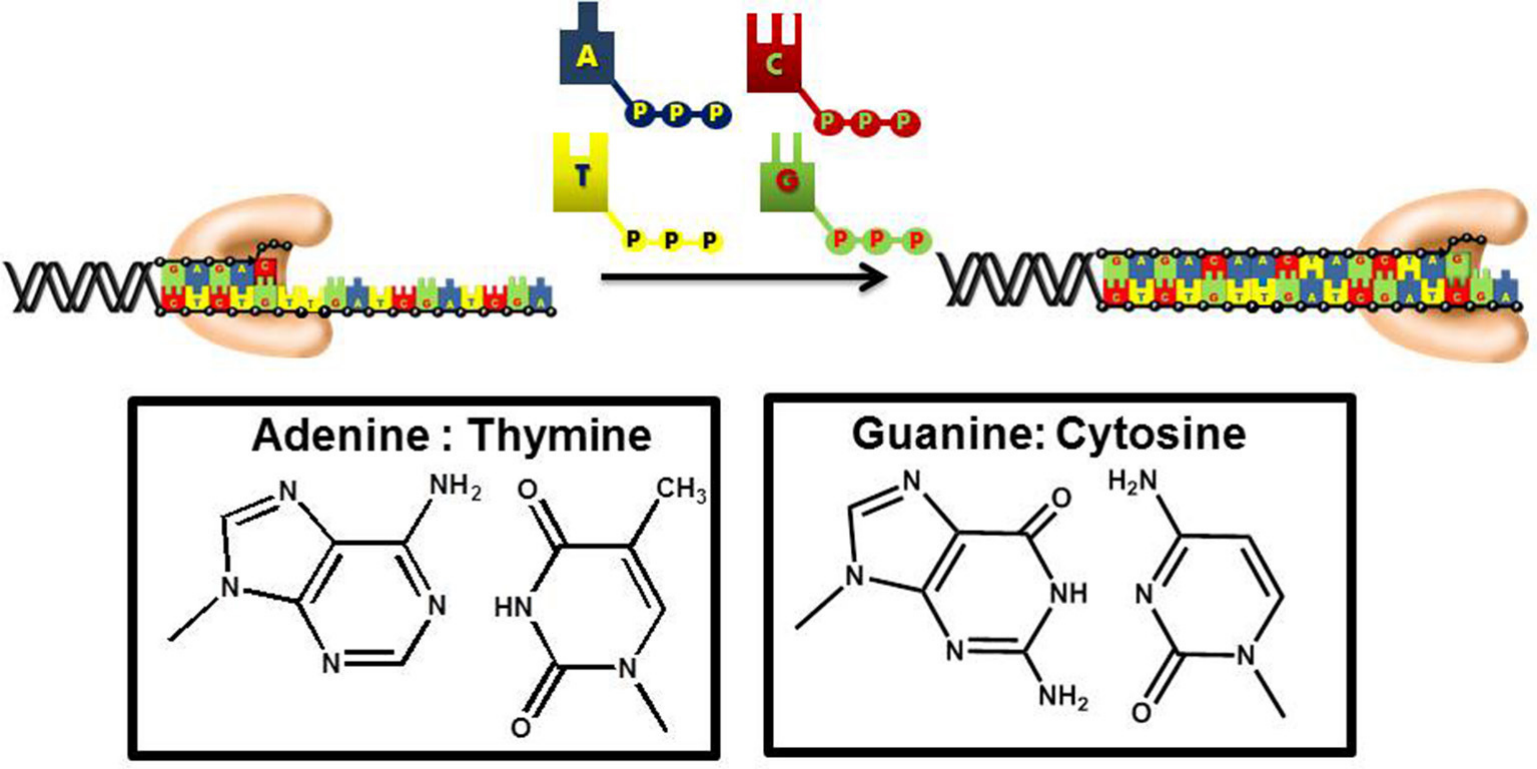
DNA polymerases use a common mechanism to synthesize DNA. During the polymerization process, a nucleotide is covalently attached to the 3′-OH group of a preexisting DNA chain serving as a primer. With most DNA polymerases, DNA is used as the template to guide each incorporation event. However, telomerase and other reverse transcriptases use or RNA as the template. Correct polymerization results in the synthesis of a DNA chain that is complementary to the template strand of DNA.

DNA replication is an essential biological pathway that produces two identical copies of an organism's genome (Garg and Burgers, 2005). In eukaryotic cells, chromosomal replication is catalyzed by a multiprotein complex termed the replicase (Kunkel and Burgers, 2008). DNA sythesis is catalyzed by DNA polymerases that incorporate mononucleotides into a primer using DNA or RNA as the template to guide each polymerization step (Figure 1). During this process, the sequence of the template varies. As such, DNA polymerases must be remarkably flexible to recognize four distinct pairing combinations of A:T, C:G, T:A, and G:C. Despite this flexibility, polymerases must also remain stringent to ensure faithful duplication of the template.

### Multiple polymerases are involved in processing nucleic acid

Humans possess at least 15 different DNA polymerases that play essential and distinct roles in chromosomal and mitochondrial replication, DNA repair, and translesion DNA synthesis, a biological process that involves the replication of damaged DNA (Hubscher et al., 2002; Shcherbakova et al., 2003). There are five DNA polymerases that participate in chromosomal DNA synthesis. These polymerases obey canonical Watson–Crick base pairing rules to catalyze both efficient and faithful DNA polymerization. In general, replicative DNA polymerases synthesize nucleic acid at incredibly high rates that approach 1,000 nucleotides per second while making only one mistake in a million opportunities (Kunkel and Bebenek, 2000; Joyce and Benkovic, 2004). Pol δ and pol ε are the two DNA polymerases most closely associated with chromosomal DNA synthesis. However, these polymerases also participate in various DNA repair pathways such as base excision repair (BER), nucleotide excision repair (NER), and mismatch repair (MMR; Downey et al., 1990; Kunkel and Burgers, 2008; Pursell and Kunkel, 2008). Pol α is a primase that synthesizes short pieces of RNA that serve as primers during the initiation of leading and lagging strand DNA synthesis (Kuchta and Stengel, 2010). Telomerase is the only eukaryotic polymerase that functions as a reverse transcriptase during the replication of telomeric regions of the chromosome (Prescott and Blackburn, 1999). Pol γ participates in the replication and repair of the mitochondrial genome (Bailey and Anderson, 2010). Pol γ, pol δ, and pol ε all possess a rigorous 3′ → 5′ exonuclease proofreading activity which contributes to the maintenance of genomic fidelity.

Several DNA polymerases are involved in completing the repair of damaged DNA. As mentioned earlier, replicative polymerases including pol δ and pol ε participate in BER, NER, and MMR. However, pol β is the primary DNA polymerase involved in BER and gap-filling synthesis during NER (Beard and Wilson, 2000). Pol λ and pol μ participate in non-homologous end joining which allows double-strand DNA breaks to be repaired (Lieber et al., 2010). Finally, B- and T-cell possess a unique DNA polymerase terminal deoxynucleotidyl transferase (TdT) that incorporates deoxynucleotides in a random fashion at double-strand DNA breaks formed during V(D)J recombination (Gucalp et al., 1991; Marshall et al., 1998). In contrast to pol δ and pol ε, DNA polymerases involved in DNA repair such as pol β, pol μ, pol λ, and TdT do not possess a 3′ → 5′ exonuclease activity.

The final group of DNA polymerases are classified as “specialized” polymerases as they are capable of replicating distinct forms of damaged DNA. Members of this family include pol η, pol ι, pol κ, pol θ, pol ψ, pol σ, pol ξ, and Rev1. Specialized DNA polymerases are similar to repair polymerases as both do not possess 3′ → 5′ exonuclease activity. The lack of proof reading activity makes these polymerases error-prone, especially when replicating undamaged DNA. Surprisngly, the majority of these polymerases are remarkably faithful when replicating damaged nucleic acid. Of all the specialized DNA polymerases identified to date, the biological function of pol η has been the most extensively characterized at the cellular and biochemical level. This polymerase is responsible for accurately replicating naturally occurring crosslinked DNA lesions such as thymine dimers (Johnson et al., 1999; Yuan et al., 2000). As described later, pol η is also very efficient at replicating DNA lesions generated by chemotherapeutic agents such as cisplatin (Alt et al., 2007). Pol ι replicates several types of modified purines (Washington et al., 2004; Nair et al., 2006; Pence et al., 2009) while Rev1 preferentially insert dCMP opposite abasic sites and most DNA lesions that involved modifications to guanine (Haracska et al., 2001, 2002b). Pol κ incorporates nucleotides opposite bulky adducts such as N^2^-acetylaminofluorene-G lesions and N^2^-benzo(a)pyrene diolepoxide-G lesions (Ohashi et al., 2000; Zhang et al., 2002). In addition, pol κ extends beyond base pairs formed by other specialized DNA polymerases during TLS (Haracska et al., 2002a). Pol ξ is similar to pol κ as that it works together with other specialized DNA polymerases to extend beyond mispairs formed by other specialized DNA polymerases (Haracska et al., 2003). The biological function and activity of other specialized DNA polymerases such as pol θ, ψ, and σ have yet to be unambiguously determined.

### Structural features of DNA polymerases

**Figure 2:**
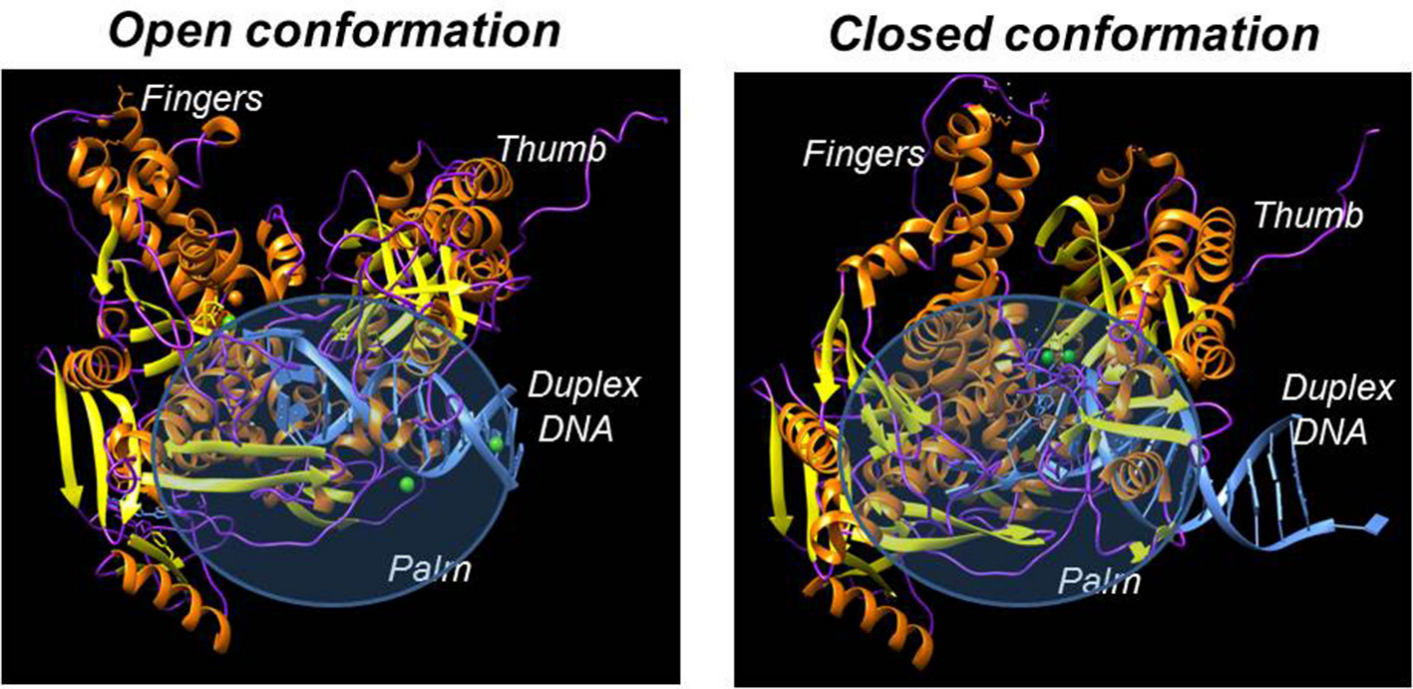
X-ray crystallographic structures of DNA polymerases reveal common structural motifs representing the palm, fingers, and thumb subdomains that play important roles in nucleotide binding and phosphoryl transfer. The left panel displays the structure of a high-fidelity DNA polymerase (bacteriophage RB69) in the “open” conformation while the right panel displays the structure of the polymerase in the “closed” conformation.

Despite having different biological functions, the overall three-dimensional structures of all DNA polymerases determined to date are remarkably similar. In general, all DNA polymerases characterized to date resemble a “right hand” possessing subdomains corresponding to a palm, thumb, and fingers (Figure 2; Steitz, 1999; Johnson and Beese, 2004; Kretulskie and Spratt, 2006). In general, the palm subdomain is highly conserved amongst all polymerases and contains two aspartates and/or glutamates that function to coordinate metal ions in the active site which are necessary for catalysis. The fingers domain plays an essential role in achieving proper nucleotide selection by interacting with the incoming dNTP and the templating base. The thumb domain is important for correctly positioning duplex DNA in the polymerization active site as well as for assisting in translocating the polymerase to the next templating base.

While structurally similar, DNA polymerases display subtle differences which significantly influence their biological functions ate the cellular level. For instance, polymerases that catalyze chromosomal replication generally possess fingers that are longer and more extended compared to specialized DNA polymerases which tend to have shorter fingers (Doublié et al., 1998; Franklin et al., 2001; Hsu et al., 2004). The longer finders of chromosomal polymerase are proposed to be important for achieving higher replication fidelity and processivity during DNA synthesis (Ling et al., 2003). In contrast, shorter fingers present of specialized DNA polymerase are believed necessary to better accommodate structurally diverse DNA lesions (Washington et al., 2003; Fleck and Schär, 2004; Steitz and Yin, 2004).

### Kinetic mechanism of DNA polymerases

**Figure 3:**
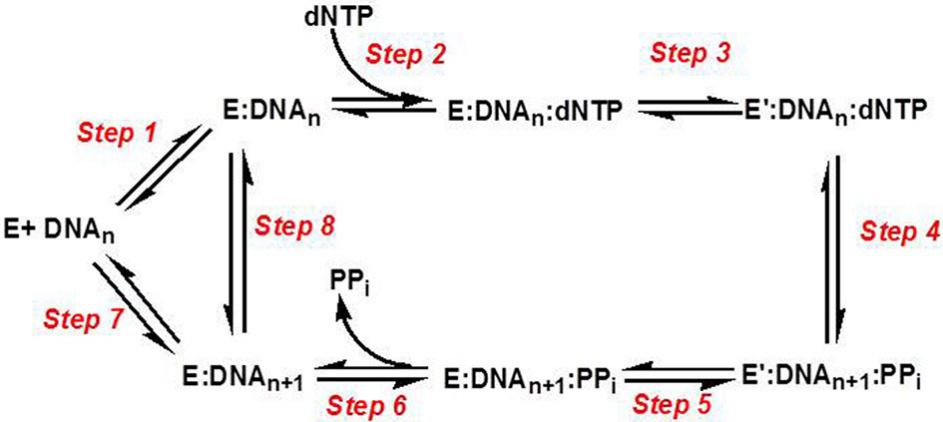
Kinetic mechanism for DNA polymerases. Individual steps along the pathway for DNA polymerization are numbered and identified as described in the text. E, polymerase; DNA_n_, DNA substrate; E′, conformational change in DNA polymerase; PP_i_, inorganic pyrophosphate; DNA_n+1_, DNA product (DNA extended by one nucleobase).

Figure 3 provides a generalized kinetic model mechanism that applies to most DNA polymerases (Mizrahi and Benkovic, 1988; Berdis, 2009; Johnson, 2010). The first step is the binding of DNA substrate to the “open” conformation of the DNA polymerase. However, dNTP binding to the “open” polymerase:DNA complex (step 2) is generally considered to be the first control point for ensuring high catalytic efficiency and polymerization fidelity during normal DNA synthesis. After binding the correct dNTP, the fingers subdomain rotates to form a “closed” conformation that orients the incoming dNTP opposite the templating base (step 3). The formation of this “closed” conformation aligns the bound dNTP into a correct geometrical orientation that allows chemistry to occur (step 4). With most high-fidelity DNA polymerases, misaligned intermediates that form as a consequence of binding an incorrect dNTP change the geometry of the polymerase's active site and causes the rate constant for the conformational change step to be reduced significantly. Lowering this rate constant provides an opportunity for the incorrect dNTP to dissociate from the Pol:DNA complex rather than to proceed through the phosphoryl transfer step. Collectively, the overall catalytic efficiency (k_pol_/K_d_) for the steps involved in correct polymerization is very large at ~10^7^ M^−1^ s^−1^. As expected, k_pol_/K_d_ values for forming mismatches (i.e., misinsertion of dATP opposite C) are typically lower by several orders of magnitude, and this reduction is caused by decreases in the binding affinity of the incoming dNTP (step 2) coupled with decreases in the rate constant for the conformational change step (step 3).

Another conformational change (step 5) occurs after phosphoryl transfer, and this step allows pyrophosphate to be released (step 6) which is coordinated with the translocation of the polymerase along DNA to the next templating position. After the translocation step, the polymerase can dissociate from the extended primer (step 7) to begin DNA synthesis on another primer template or remain bound to the elongated DNA to proceed with subsequent rounds of DNA synthesis (step 8). The ability to incorporate multiple nucleotides without dissociating from DNA defines the processivity of the polymerase. Polymerases involved in chromosomal DNA synthesis usually display high processivity as they are required to replicate thousands of base pairs per binding event. Specialized polymerases differ as they are far less processive since their involvement in replicating damaged DNA requires that they only by-pass unrepaired DNA lesions that occur sporadically throughout the genome.

In addition to polymerization activity, most high-fidelity polymerases contain an exonuclease proofreading domain that can erase potentially pro-mutagenic mismatches. The overall excision reaction is complicated since the DNA substrate must partition between the polymerase and exonuclease active sites (Reha-Krantz, 1998). After placement of primer in the exonuclease active site, the terminal nucleotide is hydrolyzed in a reaction that is generally Mg^2+^-dependent. After excision, the enzyme partitions the primer back into the polymerization domain which allows for correct DNA synthesis to be renewed without a requirement for polymerase dissociation and rebinding. This activity is important for chemotherapeutic intervention as it represents a potential mechanism of drug resistance by removing chain-terminating nucleotides from DNA.

## Chain-termination with nucleoside analogs

**Figure 4:**
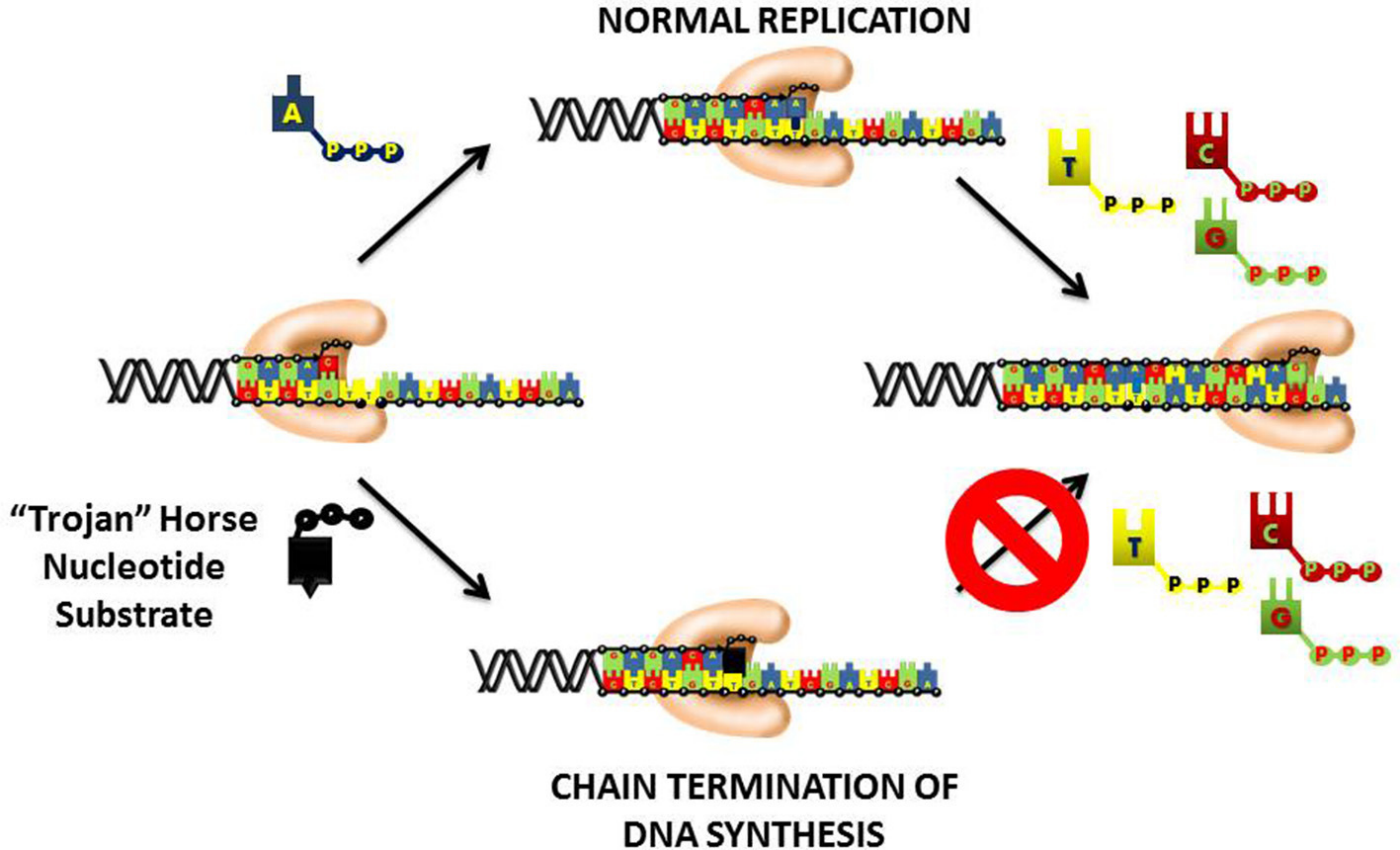
“Trojan Horse” strategy of using nucleoside analogs to inhibit DNA polymerization. The polymerase is provided with a modified nucleotide in which the 3′-OH group required for DNA elongation is missing, replaced with a halogen, or altered in configuration from a normal ribose sugar. Since the nucleobase component is left unmodified, the polymerase incorporates the nucleotide analog into DNA as efficiently as its natural counterpart. After incorporation, the nucleotide lacking a usable 3′-OH group is refractory to elongation causing the induction of apoptosis by the termination of DNA synthesis.

An important therapeutic approach to inhibit DNA replication is to commandeer the high catalytic efficiency of the chromosomal DNA polymerases into using a “suicide” nucleotide that terminates DNA synthesis (Figure 4). This “Trojan Horse” strategy is considered the major paradigm toward the rational design of nucleoside analogs that display activity as anti-cancer agents. This strategy provides a polymerase with a nucleotide analog that contains simple alterations to the deoxyribose moiety. In most instances, the 3′-OH moiety that needed to elongate DNA is substituted with non-reactive functional groups such as hydrogen (-H), halogens (Cl, F, Br, etc.), or azide (N_3_). Recently, newer approaches have generated analogs in which the entire deoxyribose moiety is replaced with an arabinose sugar that also contains a halogen in the 2′ or 3′-position. In general, the nucleobase component is left unmodified which allows the polymerase to form Watson-Crick base pairs with the templating base. As a result, the “suicide” analog is efficiently incorporated into DNA like its natural counterpart. However, the analog is devoid a usable 3′-OH group and thus produces a nucleic acid substrate that cannot be efficiently elongated. The termination is DNA synthesis causes replication fork stalling to induce apoptosis.

**Figure 5:**
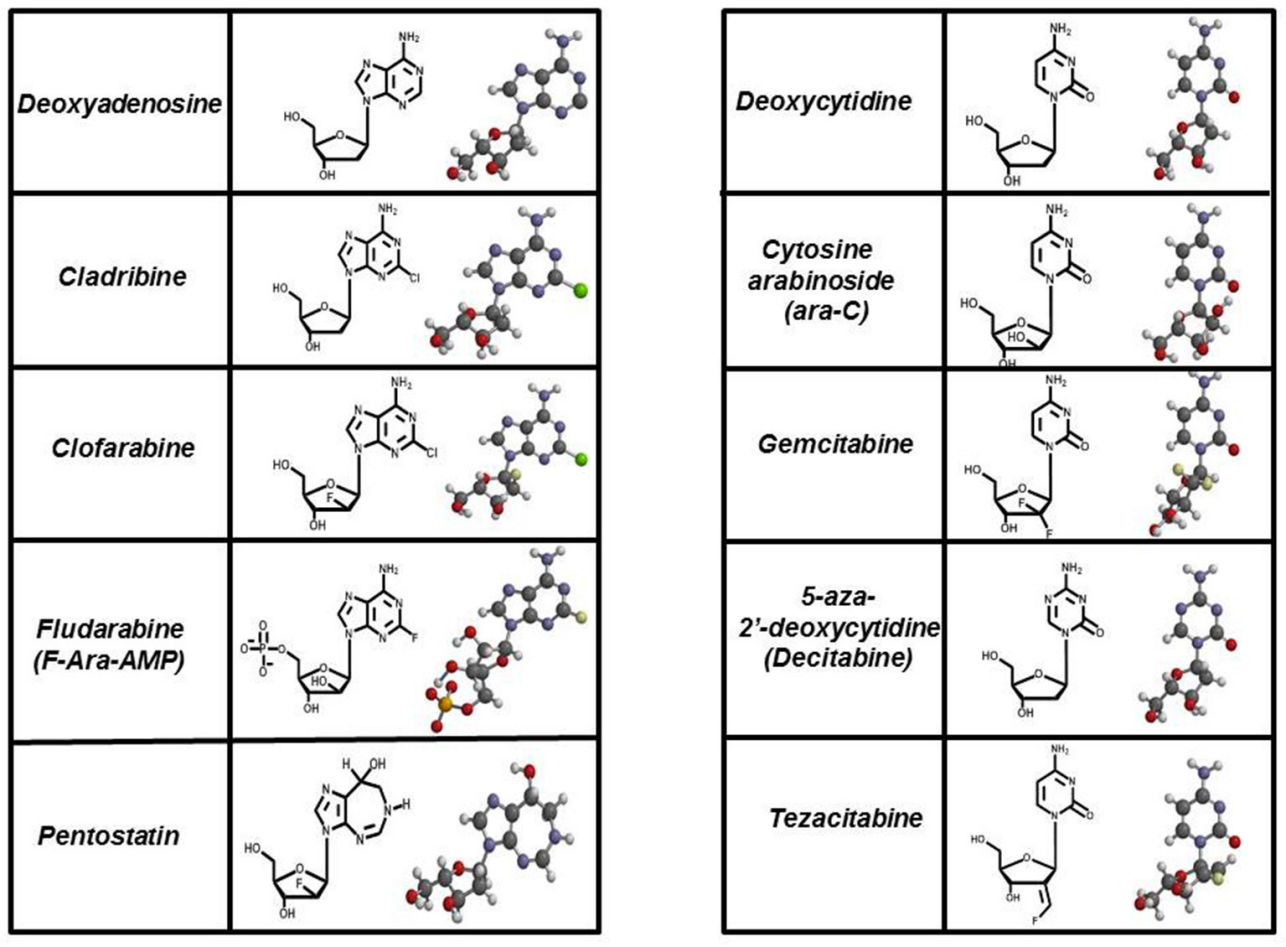
Structures of FDA approved nucleoside analogs. Purine-like nucleosides include cladribine (2-chlorodeoxyadenosine), clofarabine [2-chloro-9-(2′deoxy-2′-fluoroarabinofuranosyl)adenine], fludarabine (9-β-D-arabinoside-2-fluoroadenine), and pentostatin (2′-deoxycoformycin). Pyrimidine-like nucleosides include cytarabine [1-β-D-arabinofuranosylcytosine (Ara-C)], gemcitabine [2′,2′-difluorodeoxycytidine (dFdC)], 5-aza-deoxycytidine, and tezacitabine.

These types of “suicide” nucleotides are termed anti-metabolites and represent the largest class of antineoplastic agents used clinically (Peters et al., 1993, 2000; García et al., 2008). Currently, there are 11 nucleoside analogs that are FDA approved, and these collectively represent about 20% of all drugs used in chemotherapy (Parker, 2009). Figure 5 provides the chemical structures of several analogs that are widely used in chemotherapy. For comparison, the structures of their natural counterparts are provided as well. The most commonly used purine nucleoside analogs are fludarabine (9-β-D-arabinoside-2-fluoroadenine), cladribine [2-chlorodeoxyadenosine (2-CdA)], clofarabine [2-chloro-9-(2′deoxy-2′-fluoroarabinofuranosyl)adenine], and pentostatin (2′-deoxycoformycin). These nucleosides produce almost exclusive cytotoxic effects against hematological malignancies, most notably chronic lymphoblastic leukemia (CLL), non-Hodgkin's lymphomas, and cutaneous T-cell lymphoma (Robak et al., 2006). Commonly used pyrimidine analogs include gemcitabine and ara-C which are used to treat hematological malignancies and some solid tumors (Moysan et al., 2013).

In general, the cytotoxic effects produced by these nucleoside analogs is caused by the incorporation of their corresponding nucleoside triphosphates into DNA which results in chain termination of DNA synthesis to activate apoptosis. Discussions below focus on fludarabine (Fludara) and gemcitabine (Gemzar) as these are the two most widely used nucleoside analogs employed against cancer.

## Fludarabine

The mechanism for the incorporation of the triphosphate form of fludarabine (designated F-ara-ATP) has been extensively studied with several human DNA polymerases (Tseng et al., 1982; White et al., 1982; Parker and Cheng, 1987; Parker et al., 1988; Huang et al., 1990; Gandhi et al., 1997). While polymerases such as pol α, pol β, pol γ, and pol ε incorporate F-ara-ATP, *in vitro* studies demonstrate that the IC_50_ value for F-ara-ATP varies considerably across these enzymes. For example, F-ara-ATP inhibits pol α and pol ε most potently with *in vitro* IC_50_ values of 1.6 and 1.3 μM, respectively. The potency for F-ara-ATP is 10-fold worse with the mitochondrial polymerase, pol γ, and the DNA repair polymerase, pol β, with IC_50_ values of 44 and 24 μM, respectively. The higher potency displayed against the chromosomal DNA polymerases suggests that fludarabine exerts its therapeutic effects by inhibiting DNA synthesis during S-phase of the cell cycle.

**Figure 6:**
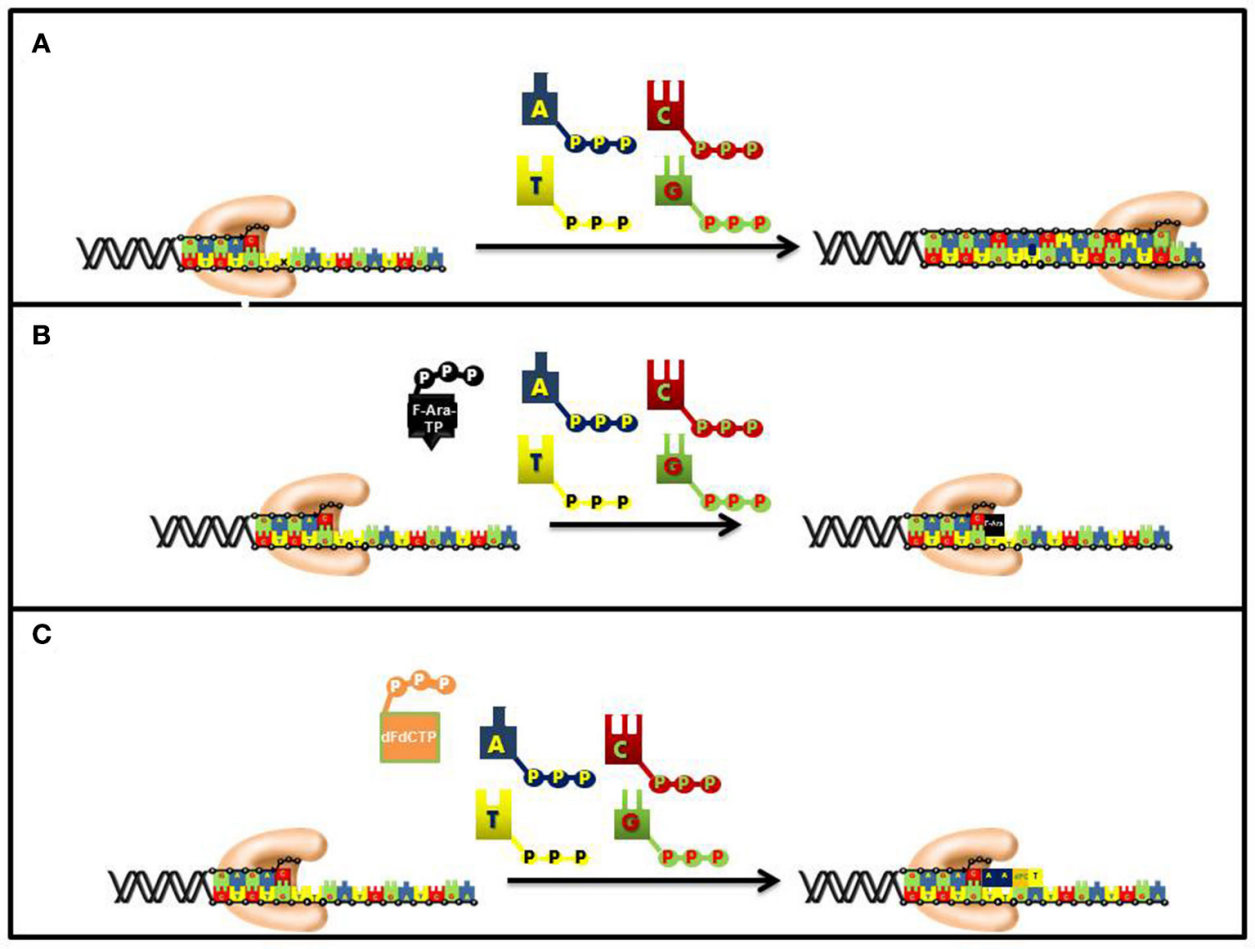
Differences in the mechanism of chain termination by gemcitabine and ara-C. After incorporation into DNA, ara-CTP terminates DNA synthesis directly at the site of incorporation while gemcitabine can be elongated by one additional nucleotide. The placement of gemcitabine at the penultimate position is termed “masked chain termination” since the terminal nucleotide masks detection and removal of gemcitabine by exonucleases or DNA repair enzymes. **(A)** Normal DNA synthesis. **(B)** Inhibition by fludarabine. **(C)** Inhibition by gemcitabine.

As expected for a competitive substrate, the inhibitory effects of F-ara-ATP can be effectively overcome through increasing concentrations of the natural nucleotide substrate, dATP. Once incorporated opposite thymine, most DNA polymerases poorly elongate beyond the modified nucleotide and this causes subsequent chain termination (Figure 6). Indeed, quantitative analyses of DNA extracted from cells incubated with tritiated nucleoside analog demonstrate that it is present at terminal positions of DNA (Spriggs et al., 1986).

Fludarabine is currently the most effective purine analog used to treat several hematological cancers including chronic lymphocytic leukemia and indolent B-cell malignancies (Hallek, 2004). Standard doses of fludarabine range between 25 and 30/mg/m^2^ given over 30 min for five consecutive days. Under these conditions, a plasma concentration of 3 μM for the analog is achieved within 30 min. Peak concentrations of the active metabolite, F-ara-ATP, are about 4 h post-infusion (Malspeis et al., 1990).

## Gemcitabine

The synthetic pyrimidine analog, gemcitabine [2′, 2′-difluorodeoxycytidine (dFdC)], differs from deoxycytidine by the addition of two fluorine atoms in the geminal configuration at the 2′-position of sugar (Figure 5). Gemcitabine produces a wide spectrum of anti-cancer activities against hematological cancers and solid tumors. The triphosphate form of gemcitabine, dFdCTP, functions as a substrate for a number of DNA polymerases involved in chromosomal replication, DNA repair, and translesion DNA synthesis (Huang et al., 1991; Jiang et al., 2000). For example, the IC_50_ values for dFdCTP are 11 and 14 μM for pol α and pol ε, respectively. Likewise, DNA primer extension assays performed using *in vitro* analyses show that there is direct competition between FdCTP and dCTP for insertion opposite guanine. After dFdC is incorporated, the modified pyrimidine can be elongated one additional nucleotide before DNA synthesis is terminated (Figure 6). This unique activity contrasts that of F-ara-TP which typically terminates DNA synthesis directly at the site of its incorporation. The unique method of inhibiting DNA synthesis by dFdCMP is coined “masked chain termination” as the addition of an extra nucleotide essentially hides the incorporated dFdCTP from various enzymes that could excise the pyrimidine analog from DNA to reverse its effect of DNA synthesis (Plunkett et al., 1995).

Gemcitabine is used as a monotherapeutic agent in the treatment of certain leukemias, lymphomas, and metastatic pancreatic cancer (Eckel et al., 2006). However, gemcitabine is more frequently combined with platinum drugs such as cisplatin and oxaliplatin (Hoff and Fuchs, 2003; Ozols, 2005; Sehouli, 2005; Chua and Cunningham, 2006; Richardson et al., 2008) to treat solid cancers such as non-small-cell lung, bladder, ovarian, and breast cancers (Lorusso et al., 2005; Silvestris et al., 2008). The reason for combining gemcitabine with platinum agents is based on cell-based data demonstrating that the combination of drugs produces a synergistic cell-killing effect. At the clinical level, treatment with oxaliplatin can cause serious complications such as peripheral neurotoxicity and nephrotoxicity (Meliani et al., 2003). In contrast, gemcitabine is a well-tolerated drug as it produces mild side effects such as moderate myelosuppression, asthenia, and nausea/vomiting (Teusink and Hall, 2010). As a result, gemcitabine is used to sensitize the effects of platinum drugs so that lower doses of platinum-based DNA damaging agents can be administered acutely and cumulatively to avoid serious side effects.

## DNA damaging agents

Another major strategy in chemotherapy is to use DNA damaging agents to inhibit processive DNA polymerases. Since DNA damaging agents are very electrophilic, they effectively react with nucleophilic moieties on DNA to significantly modify the hydrogen-bonding potential and structure of nucleic acid. In most instances, the formed DNA lesion acts as a physical barrier and hinders the movement of a DNA polymerase to inhibit DNA synthesis. In other cases, the change in hydrogen-bonding information on DNA tends to increase the frequency of misincorporation events to subsequently enhance the occurrence of pro-mutagenic DNA synthesis. The mismatches that are formed become excellent substrates for enzymes involved in various DNA repair pathways which can either correct the damaged DNA or cause cell death. The cellular effects of temozolomide (TMZ), a monofunctional alkylating agent, represent an excellent example of this phenomenon. TMZ produces cytostatic and cytotoxic effects primarily through the non-enzymatic methylation of DNA. Specifically, TMZ creates a number of DNA lesions including N^3^-methyladenine, O^6^-methylguanine, and N^7^-methylguanine, the most commonly formed DNA adduct (Gates et al., 2004). Methylation at the N7 position of guanine produces a more toxic DNA lesion, termed an abasic site, which forms by the spontaneous depurination of the methylated base (Friedman et al., 2000). Since abasic sites lack Watson-Crick coding information, they are classified as non-instructional DNA lesions and typically inhibit the synthetic activity of most high-fidelity DNA polymerases (Shcherbakova et al., 2003). In contrast, alkylation of the *O*^6^ position of guanine changes its hydrogen-bonding potential which increases the frequency of misincorporation events (Woodside and Guengerich, 2002). The resulting mispair that results from the misincorporation of dTMP opposite *O*^6^-methylguanine activates the MMR pathway to ultimately induce apoptosis (Koç et al., 1996).

There are a large number of chemotherapeutic agents that exert their effects by damaging DNA as well. For example, one of the most used therapeutic modalities against solid tumors is ionizing radiation which creates radicals that inflict damage on nucleic acid (Santivasi and Xia, 2014). Doxorubicin is classified as a tetracycline antibiotic which intercalates into DNA to produce a variety of cellular effects (Pommier et al., 2010). First, the interaction with DNA inhibits the progression of topoisomerase II, an enzyme involved in relaxing supercoiled DNA that forms during replication and transcription. In addition, the quinone moiety of doxorubicin enhances free radical production in an oxygen-dependent manner to cause DNA damage. In both cases, the end result is the production of double-strand DNA breaks (DSBs) which inhibit DNA synthesis. Etoposide is similar in function as it forms a complex with DNA and topoisomerase II. The formation of this ternary complex inhibits the ability of topoisomerase II to re-ligate DNA, and this ultimately creates DSBs (Meresse et al., 2004). Camptothecin, a natural product isolated from the tree, Camptotheca acuminate, is a quinolone alkaloid that also creates DSBs by inhibiting the activity of topoosiomerase I (Liu et al., 2000). Unfortunately, camptothecin produces a number of adverse side effects in cancer patients and as such is not widely used clinically. However, two modified analogs of camptothecin (topotecan and irinotecan) display more favorable pharmacodynamic behavior and are used to treat several types of solid tumors (Mathijssen et al., 2002). Similar to the parental compound, topotecan and irinotecan exert their cytotoxic effects by generating DSBs.

With all of these agents, the DSBs that are formed directly inhibit DNA synthesis since these non-instructional lack Watson-Crick coding information (Boulton et al., 2000). DSBs can be repaired by non-homologous end-joining (NHEJ) and homologous recombination (HR). NHEJ and HR use different DNA polymerases to efficiently and completely repair formed DSBs. Cisplatin, chlorambucil and cyclophosphamide represent another type of DNA damaging agent that is widely used to treat hematological and solid tumors (Passerini and Ponticelli, 2003; Anders et al., 2006). These agents are bifunctional alkylating agents that can create crosslinks and/or bulky adducts that produce physical barriers which inhibit DNA synthesis. By stalling DNA synthesis, these lesions generate single-stranded DNA breaks (SSBs) and DSBs that can cause cell death if left unrepaired.

## Combination therapies

There is substantial clinical evidence supporting a strategy for combining nucleoside analogs with DNA damaging agents. As indicated earlier, gemcitabine is frequently combined with platinum drugs such as cisplatin and oxaliplatin to treat ovarian and pancreatic cancer (Hoff and Fuchs, 2003; Ozols, 2005; Sehouli, 2005; Chua and Cunningham, 2006). Several pre-clinical studies have examined the underlying mechanism for how gemcitabine synergizes the cytotoxic effects of platinum-based drugs. Using the ovarian cancer cell line, A2780, as a model, Jensen et al. showed that gemcitabine combined with cisplatin caused an increase in the amount of platinum-DNA adducts compared to cisplatin treatment alone (Jensen et al., 1997). The higher number of DNA adducts appeared to result from a decrease in DNA repair that was caused by the inhibition of cellular exonucleases such as excision repair cross-complementation group 1 (ERCC1). However, other models such as the inhibition of specialized DNA polymerases by gemcitabine have also been invoked (Chen et al., 2008). This model is based on evidence showing that pol η-deficient cells are more sensitive to the combination of gemcitabine and cisplatin compared to normal fibroblast that are pol η-proficient. In addition, pol η-deficient cells are ~10-fold more sensitive to the combined treatment of gemcitabine and cisplatin compared to treatment with cisplatin alone.

Surprisingly, attempts to combine other nucleoside analogs such as fludarabine (Fludara) and cladrabine (Leustatin) with DNA damaging agents have proven unsuccessful. For example, a study performed by Rai et al. was discontinued since patients receiving fludarabine and chlorambucil showed evidence for excessive hematological toxicity with no improvement in overall response compared to fludarabine monotherapy (Rai et al., 2000). A similar study using chlorambucil with escalating doses of fludarabine in patients with CLL also showed high levels of hematological toxicity (Weiss et al., 1994). Identical complications have been experienced in patients receiving cladribine and chlorambucil (Tefferi et al., 1994). The reason for the onset of these hematological toxicities may reflect a lack of selectivity exhibited by these purine nucleosides. In this case, the higher potency of fludarabine against replicative DNA polymerases may cause non-specific killing by placing a high burden on DNA replication and DNA repair in healthy cells.

## Drug resistance caused by translesion DNA synthesis

**Figure 7:**
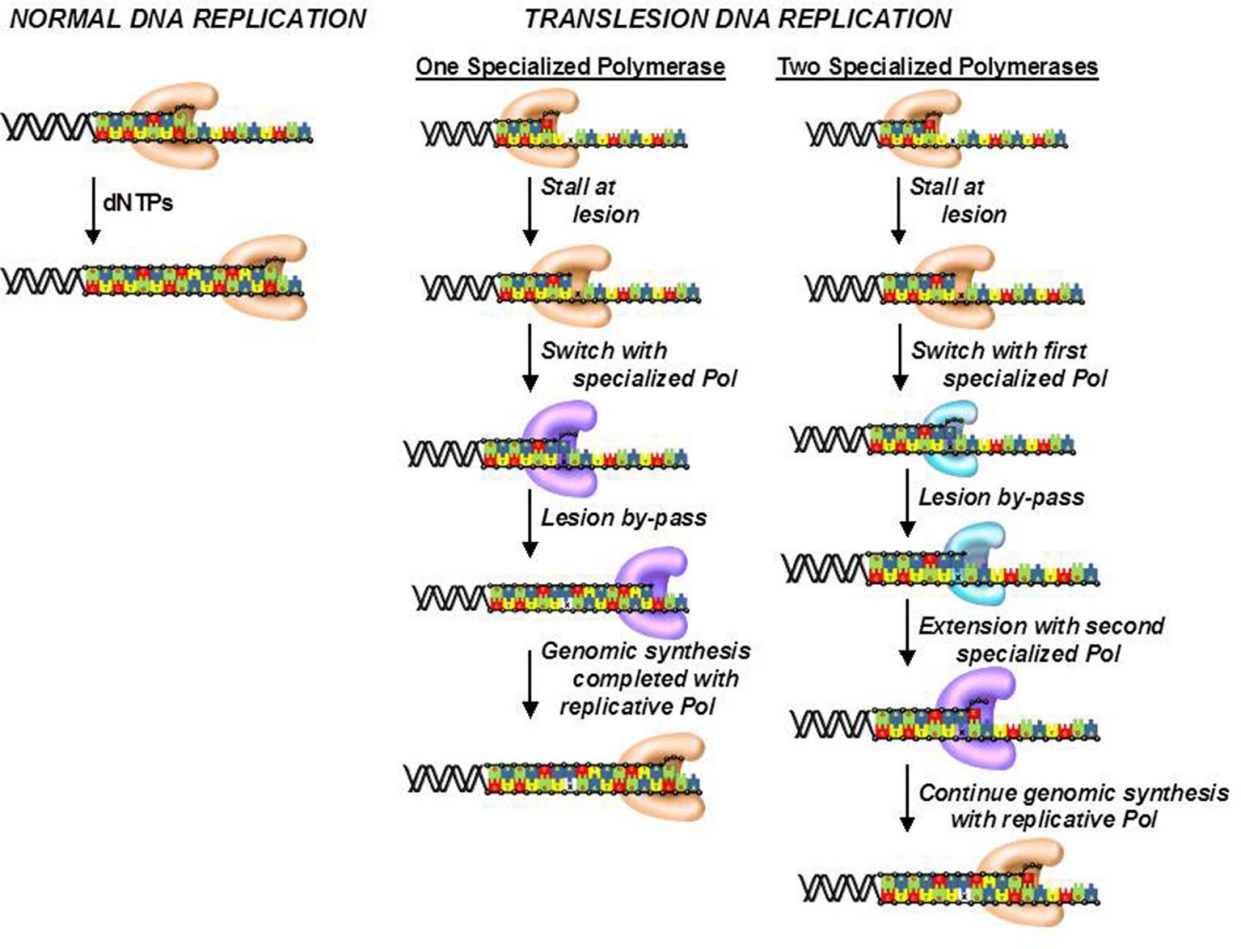
Models for the efficient bypass of DNA lesions during translesion DNA synthesis. After encountering a DNA lesion, a replicative DNA polymerase incorporates a nucleotide opposite it but is unable to extend beyond the lesion. A specialized DNA polymerase extends beyond the lesion. Once the lesion is bypassed, the specialized polymerase is replaced by the replicative polymerase to resume processive DNA synthesis. In some cases, the replicative polymerase stalls at the DNA lesion and is unable to incorporate a nucleotide opposite the adduct. As a result, a specialized polymerase is recruited to the DNA lesion to incorporate a nucleotide opposite the lesion. A different specialized polymerase is then recruited for extension beyond the DNA lesion.

Although, cells possess several DNA repair pathways, there are situations in which DNA lesions are not detected and persist to block DNA synthesis catalyzed by high-fidelity DNA polymerases. To avoid this, cells use the unique activity of various specialized DNA polymerases to replicate unrepaired lesions in a process termed translesion DNA synthesis (TLS). As expected, the coordination of TLS activity at the cellular level is remarkably complex, and much of this complexity arises from the number of DNA polymerases that can replicate the various types of DNA lesions produced by endogenous and exogenous agents. Despite these complexities, there are two general models that describe how DNA polymerase activities are coordinated during TLS (Figure 7; Friedberg et al., 2005; McCulloch and Kunkel, 2006). In one model, a replicative DNA polymerase encounters a DNA lesion and incorporates a nucleotide opposite the DNA lesion. Since replicative polymerases display high-fidelity, they generally do not extend beyond the DNA lesion. This causes stalling of the replication fork which serves as a signal to enlist the activity of a specialized DNA polymerase such as pol κ or pol ξ to incorporate nucleotides beyond the lesion. Once the damaged DNA is by-passed, the specialized DNA polymerase is displaced by the replicative enzyme to continue DNA synthesis on the remainder of undamaged DNA. This model could occur with DNA lesions such as O^6^-methylguanine and 8-oxo-guanine.

The second model differs slightly as the replicative polymerase is unable to incorporate a nucleotide opposite the lesion. Instead, one or more specialized DNA polymerases are used to incorporate a dNTP opposite the lesion. Depending upon the nature of the damaged DNA, the specialized DNA polymerase can extend beyond it as well. However, a different specialized polymerase such as pol ξ is often recruited to further elongate beyond the DNA lesion. Once the lesion is effectively by-passed, a replicative DNA polymerase replaces the extender polymerase and continues processive DNA synthesis on the remainder of the undamaged DNA. This scenario likely occurs with crosslinked or large bulky DNA lesions such as thymine dimers and cisplatinated DNA.

A number of retrospective clinical trials have been recently performed to examine possible correlations between patient responses to DNA damaging agents with the expression level of certain specialized DNA polymerases. Several studies have identified a group of distinct DNA polymerases that play important roles in modulating patient responses to certain chemotherapeutic agents. In particular, overexpression of DNA polymerases such as pol β, pol η, pol λ, and pol ι is observed in many different types of tumors (Albertella et al., 2005; Tan et al., 2005; Yoshizawa et al., 2009).

These specialized DNA polymerases also play important roles in defining how patients respond to certain chemotherapeutic agents. For example, pol η can extend beyond cisplatin-DNA lesions, and overexpression of this specialized DNA polymerase causes resistance to cisplatin in cancer cell lines whereas downregulation causes increased cellular sensitivity to cisplatin (Nivard et al., 2005; Chen et al., 2006). Higher mRNA expression of pol η is associated with poor outcomes in patients with non-small-cell lung cancer and is also associated with shorter survival times in patients receiving platinum drugs (Ceppi et al., 2009). Similar observations are seen with other specialized DNA polymerases such as pol ι which is overexpressed in breast cancer cells and found to be upregulated in ~30% of glioma tumors (Yang et al., 2004) Overexpression of pol ι also appears to be clinically relevant as patients with pol ι-positive gliomas had shorter survival rates (Yang et al., 2004).

## Emerging areas

These examples suggest that selectively inhibiting one or more specialized DNA polymerases may provide a new strategy to combat clinical complications associated with unregulated TLS activity. In fact, inhibiting the replication of DNA lesions produced by anti-cancer agents may generate a number of positive effects in cancer patients that undergo chemotherapy. First, inhibiting TLS activity would likely increase the cytotoxic effects of DNA damaging agents and potentiate their effectiveness, especially in cancer cells that are defective in DNA repair. The benefit of potentiation is that lower doses of DNA damaging agents could be administered, thus reducing the risk of potential side effects. In addition, inhibiting TLS activity would combat drug resistance caused by the replication of damaged DNA. Finally, preventing pro-mutagenic DNA synthesis could hinder cancer recurrence caused by mutagenesis.

Efforts in our laboratory have focused on developing artificial nucleosides that are efficiently utilized by specialized DNA polymerases during the replication of lesions generated by DNA damaging agents. One DNA lesion that plays an important therapeutic role is the abasic site, a non-instructional form of DNA damage that is produced by several anti-cancer agents including TMZ and cyclophosphamide. To inhibit the replication of this lesion, we generated an artificial nucleotide, designated 3-ethynyl-5-nitroindolyl-2′-deoxyriboside triphosphate (3-Eth-5-NITP), that is a more efficient substrate than dATP, the natural nucleotide that is preferentially utilized by several human DNA polymerases during TLS (Motea et al., 2012). *In vitro* kinetic approaches compared the ability of pol δ, the high-fidelity polymerase involved in chromosomal replication and pol η, a specialized DNA polymerase, to incorporate dATP and 3-Eth-5-NITP opposite an abasic site (Choi et al., 2017). Our studies showed that pol η is 500-fold more efficient at incorporating dATP opposite the non-instructional lesion compared to the high-fidelity polymerase, pol δ. This large difference verifies that pol η contributes significantly to the error-prone replication of this lesion. More importantly, we demonstrated that pol η utilizes 3-Eth-5-NITP ~30-fold more efficiently than dATP when replicating an abasic site. Furthermore, this artificial analog blocks extension beyond the lesion and terminates pro-mutagenic DNA synthesis. Cell-based studies demonstrate that the corresponding artificial nucleoside (3-Eth-5-NIdR) potentiates the effects of certain DNA damaging agents that produce abasic sites (Choi et al., 2017). Using acute lymphoblastic leukemia (ALL) cells as a model, we showed that co-treatment with TMZ and sub-lethal doses of 3-Eth-5-NIdR results in a synergistic increase in cell death. This synergism in apoptosis was caused by inhibiting TLS activity, and this was confirmed as the levels of 3-Eth-5-NITP in genomic DNA were higher in cells treated with 3-Eth-5-NIdR and DNA damaging agent compared to cells treated with 3-Eth-5-NIdR alone. We are currently testing the efficacy of combining 3-Eth-5-NIdR with TMZ in several xenograft mouse models of human cancer to demonstrate proof-of-concept for this strategy. Preliminary data from these *in vivo* studies look very promising, and the theranostic capabilities of 3-Eth-5-NIdR could usher in a new strategy in precision-based therapies against cancer.

## Author contributions

The author confirms being the sole contributor of this work and approved it for publication.

### Conflict of interest statement

The author declares that the research was conducted in the absence of any commercial or financial relationships that could be construed as a potential conflict of interest.
